# Theoretical Investigation of the Material Usage During On-Bead Enrichment of Post-Translationally Modified Peptides in Suspension Systems

**DOI:** 10.3390/molecules30153245

**Published:** 2025-08-02

**Authors:** Kai Liu, Yuanyu Huang, Thomas Huang, Pengyuan Yang, Jilie Kong, Huali Shen, Quanqing Zhang

**Affiliations:** 1 Department of Chemistry, Fudan University, Shanghai 200433, China; 20110220123@fudan.edu.cn (K.L.); 18110220040@fudan.edu.cn (Y.H.); pyyang@fudan.edu.cn (P.Y.); jlkong@fudan.edu.cn (J.K.); 2 Institute for Integrative Genome Biology, Proteomics Core, University of California Riverside, Riverside, CA 92521, USA; thomas.huang001@email.ucr.edu; 3 Institutes of Biomedical Sciences of Shanghai Medical School, Fudan University, Shanghai 200032, China

**Keywords:** post-translational modifications, theoretical investigation, glycopeptide enrichment, phosphopeptide enrichment, material dosage

## Abstract

Over the past decade, the number and diversity of identified protein post-translational modifications (PTMs) have grown significantly. However, most PTMs occur at relatively low abundance, making selective enrichment of modified peptides essential. To address this, we developed a thermodynamic model describing the free beads enrichment in suspension enrichment process and derived a theoretical relationship between material dosage and analyte recovery. The model predicts a non-linear trend, with enrichment efficiency increasing up to an optimal dosage and declining thereafter—a pattern confirmed by experimental data. We validated the model using centrifugation-based enrichment for glycosylated peptides and magnetic-based enrichment for phosphorylated peptides. In both cases, the results aligned with theoretical predictions. Additionally, the optimal dosage varied among peptides with the same modification type, highlighting the importance of tailoring enrichment strategies. This study provides a solid theoretical and experimental basis for optimizing PTMs enrichment and advancing more sensitive, accurate, and efficient mass spectrometry-based proteomic workflows.

## 1. Introduction

Post-translational modifications (PTMs) are covalent processing events that change the properties of a protein by proteolytic cleavage or by addition of a modifying group to one or more amino acids. PTMs of a protein can determine its activity state, localization, turnover, and interactions with other proteins [1]. Furthermore, PTMs play key roles in regulating cell signaling and physiology in both normal and cancer cells [2]. As many as 300 PTMs of proteins are known to occur physiologically [3]. The number of human protein isoforms generated by alternative splicing alone has been estimated to be around 100,000, pushing the total number of proteoforms generated by splicing and PTMs to the tens of millions at least [4]. However, the most-studied non-proteinaceous PTMs remain enzyme-catalyzed phosphorylation, acetylation, methylation, glycosylation, and palmitoylation, as well as the nonenzymatic glycation and nitrosylation [5]. PTMs can also consist of separate polypeptides or protein domains conjugated via isopeptide bonds. In addition to classical ubiquitination and SUMOylation, modifications by other ubiquitin-like molecules (UBIs) have increasingly also gained attention [6,7]. Many other PTMs currently lack mature technologies for their identification and analysis. As a result, we probably still do not realize the full extent and functional importance of protein modifications in the workings of the cell.

Mass spectrometry (MS) is a central technology in the protein chemist’s toolkit, enabling site mapping and quantification of chemical modifications on proteins, as well as detection of new types of structures [8,9]. Emerging MS-based proteomics approaches are powerful alternative methods used to characterize PTMs [10,11]. With different dissociation methods, such as HCD, ETD, and UVPD enabling high efficiency PTMs identification [12,13]. With the advancement of mass spectrometry technology, detection sensitivity has significantly improved, making it possible to identify PTMs at the single-cell level [9,14,15,16]. However, biological samples (such as body fluids or tissues) contain a large number of diverse proteins, and the abundance distribution of these proteins is highly uneven, with differences reaching up to 11 orders of magnitude [17,18,19]. Moreover, the presence of various PTMs [20,21,22] further increases the complexity of the sample. This high level of complexity presents significant challenges in the selection and optimization of analytical methods, prompting researchers to continually explore more efficient strategies in order to obtain more comprehensive and biologically meaningful information.

The introduction of various separation and enrichment techniques has effectively enhanced the sensitivity and specificity of overall PTMs research strategies [23,24]. For example, multidimensional chromatography [25,26] improves sample resolution through multistage separation, while novel sample pretreatment methods facilitate the selective enrichment of target compounds, thereby improving detection efficiency. In recent years, nanometer-scale separation media with high selectivity and capacity have made significant progress in the fields of analytical science and biomedical research. These nanomaterials, owing to their excellent specific surface area, tunable pore structures, and efficient adsorption capabilities, play a critical role in the separation and enrichment of complex samples. The integration of these techniques not only improves the capability for trace-level analysis in omics research but also offers more precise and efficient approaches for biomarker discovery, environmental pollutant detection, drug metabolism studies [27,28,29], and targeted sample enrichment strategies [30,31]. Currently, the optimal methods for enriching PTMs are primarily based on experimental approaches or partial quantification using specific samples [32,33,34]. Therefore, when enriching targets with extremely low abundance and fixed concentrations, the optimized material dosage obtained is usually an empirical value rather than a theoretical one.

Magnetic solid-phase extraction (MSPE) is a promising and efficient sample pretreatment technique that uses magnetic materials for rapid enrichment, especially suitable for complex samples [35,36]. Compared to traditional SPE, MSPE avoids issues like clogging and simplifies operation. However, theoretical understanding—particularly the relationship between material dosage and analyte concentration—is limited. This study develops a physical model to describe these interactions. We validated the model through a series of controlled enrichment experiments. The results demonstrated strong agreement between theoretical predictions and experimental outcomes. This work not only deepens our understanding of the MSPE mechanism but also provides a practical framework for improving its efficiency and selectivity in complex biological samples, thereby advancing its application in proteomics and broader omics research.

## 2. Results

### 2.1. Thermodynamic Characteristics of the MSPE Separation and Enrichment Process

In suspension-based enrichment systems utilizing free beads, the interaction between magnetic materials and target molecules involves key thermodynamic and kinetic factors such as adsorption equilibrium, binding energy, and diffusion rate. Studying the thermodynamics of this process is essential for understanding selective adsorption mechanisms and optimizing enrichment strategies across various sample types.

This research focuses on modeling the enrichment mechanism of magnetic materials. We first establish a thermodynamic model based on physicochemical principles to identify key influencing factors. The model is then validated through experimental data, and critical parameters are optimized to bridge theoretical predictions with practical applications. This approach provides a scientific basis for advancing magnetic enrichment techniques, particularly in high-throughput proteomics.

**Figure 1:**
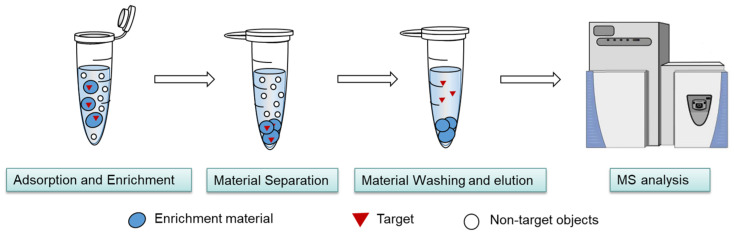
Basic workflow of free beads enrichment in suspension. The enrichment material is mixed with the complex sample containing the target analyte and allowed to interact fully. Afterward, the enriched material is separated from impurities and washed multiple times to reduce nonspecific adsorption. Finally, the target analyte is eluted from the material and subjected to detection and identification.

As shown in Figure 1, the general workflow of in suspension enrichment using free beads typically involves the following steps:

(1) Adsorption and Enrichment: Add a mass $m_{0}$ of magnetic separation material to a sample solution with volume $V_{0}$ and initial target concentration $c_{0}$. The material is uniformly dispersed to adsorb and enrich the target compound.

(2) Material Separation: Use an external magnetic field (e.g., a magnet or electromagnet) or centrifugation to separate the target-bound magnetic material from the solution.

(3) Material Washing and elution: Wash the material with a mild elution solvent to remove nonspecifically bound substances, enhancing enrichment selectivity. Finally, elute the enriched target from the material using an elution solvent with volume $V_{1}$.

In practical applications, the target compounds for enrichment are often present at low or trace concentrations, meaning the solution typically behaves as a dilute solution. Under these conditions, the adsorption process does not lead to overloading of the material. Let $\alpha_{1}$ denote the amount of solute that can be adsorbed per unit mass of material under these conditions, and $S_{0}$ represent the specific surface area per unit mass of the material. Most enrichment materials are either non-porous or have surface porosity, while through-pore structures are rare. Therefore, it is reasonable to assume that adsorption primarily occurs on the material surface. Additionally, under experimental conditions, adsorption is assumed to reach equilibrium rapidly. Based on these assumptions, the mass balance equation for the first-step enrichment process can be expressed as follows:

(1) $$V_{0}c_{0}=V_{0}c_{1}+m_{0}\alpha_{1}$$

In this context, $c_{1}$ represents the concentration of the solute in the solution when equilibrium is reached, while the equilibrium constant k is defined as follows:

$$k=\frac{c_{1}}{\alpha_{1}/S_{0}}=\frac{c_{1}S_{0}}{\alpha_{1}}$$

(2) $$c_{1}=\frac{k\alpha_{1}}{S_{0}}$$

Substituting Equation (2) into Equation (1) yields the following:

$$V_{0}c_{0}=V_{0}k\alpha_{1}/S_{0}+m_{0}\alpha_{1}=\alpha_{1}(V_{0}k/S_{0}+m_{0})$$

(3) $$\alpha_{1}=\frac{V_{0}c_{0}}{V_{0}k/S_{0}+m_{0}}$$

By combining Equation (3) with Equation (1), we can express the amount of solute adsorbed from the solution in the second step of the enrichment process as follows:

(4) $$m_{0}\alpha_{1}=\frac{V_{0}c_{0}}{V_{0}k/m_{0}S_{0}+1}$$

The mass balance equation for the third step is as follows:

(5) $$m_{0}\alpha_{1}=V_{1}c+m_{0}\alpha_{2}$$

In this scenario, $\alpha_{2}$ denotes the quantity of the target solute that remains on each unit of adsorbent material after the elution process. Assuming the equilibrium constant for this process is $k_{1}$, it can be expressed as follows:

$$k_{1}=\frac{c}{\alpha_{2}/S_{0}}=\frac{cS_{0}}{\alpha_{2}}$$

(6) $$\alpha_{2}=cS_{0}/k_{1}$$

Substituting Equation (6) into Equation (5) results in the following:

(7) $$m_{0}\alpha_{1}=V_{1}c+m_{0}cS_{0}/k_{1}=c(V_{1}+m_{0}S_{0}/k_{1})$$

Further combining Equation (7) with Equation (4), we obtain the following:

(8) $$c/c_{0}=\frac{V_{0}k_{1}/S_{0}m_{0}}{\left(V_{0}k/S_{0}+m_{0}\;)(V_{1}k_{1}/S_{0}+m_{0}\right)}$$

Equation (8) represents the key relationship derived from our study, useful for investigating the optimal conditions of the enrichment process and the effects of various operational parameters on enrichment efficiency. Once the material’s characteristics are established, the equilibrium constant is influenced only by the properties of the solvent, such as its temperature and pressure. Therefore, the volume in the system, the equilibrium constant, and the specific surface area of the material are either known or can be calculated, making them computable constants. If defining $V_{0}S_{0}/k_{1}=A,\;V_{0}k/S_{0}=B,\;V_{1}k_{1}/S_{0}=C$. Where the units of A, B, and C are in micrograms (μg), and their values are either known or can be calculated. Then, Equations (5)–(9) can be transformed into the following:

$$c/c_{0}=\frac{Am_{0}}{\left(B+m_{0})(C+m_{0}\right)}$$

Which is

$$c_{0}/c=\frac{\left(B+m_{0})(C+m_{0}\right)}{Am_{0}}$$

$$c_{0}/c=\frac{\left(B+m_{0})(C+m_{0}\right)}{Am_{0}}=\frac{1}{A}m_{0}+\frac{BC}{A}\frac{1}{m_{0}}+\frac{B+C}{A}$$

If defining 1/A=D,  BC/A=E,  (B+C)/A=F. Where the units of *D is 1/μg*, *E is μg*, and *F* is dimensionless. Their values are either known or can be calculated. So, we have

$$c_{0}/c=Dm_{0}+\frac{E}{m_{0}}+F$$

**Figure 2:**
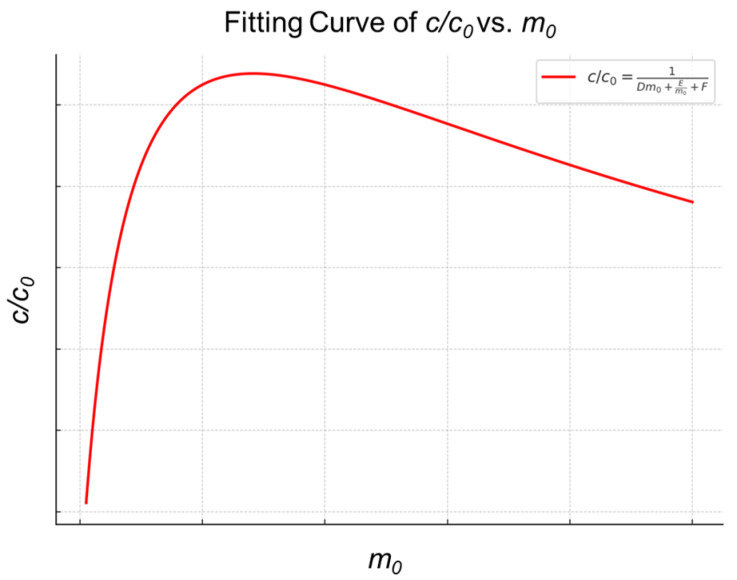
Simulated Relationship graph between $c/c_{0}$ and $m_{0}$.

When the volume of the elution solvent is the same as the initial sample volume, c/$c_{0}$ is equivalent to the mass recovery yield of the enrichment. The enrichment yield $=c/c_{0}=\frac{1}{c_{0}/c}$. Graphing m_0_ against Y, we obtain the results depicted in Figure 2. Based on this curve, we can effectively determine the optimal amount of material required to achieve the highest recovery rate during the enrichment.

As shown in Figure 2, the plot of $m_{0}$ versus $c_{0}/c$ exhibits a peak, indicating the point at which the recovery of the target compound is maximized. This inflection point can serve as a basis for optimizing enrichment conditions. Thermodynamic and kinetic studies of the enrichment process confirm that the relationship between the amount of enrichment material and the target analyte is not simply linear. Instead, there exists a non-linear trend characterized by an initial rise followed by a decline. When the target concentration and solution volume are fixed, increasing the amount of enrichment material initially improves enrichment efficiency, but beyond a certain point, it begins to decline. This model is based on a dilute solution system with uniformly suspended enrichment materials; therefore, it cannot be applied to other enrichment systems, such as chromatography-based enrichment approaches.

### 2.2. Experimental Validation of the Material Dosage-Analyte Relationship Trend

#### 2.2.1. Highly Selective Enrichment of *N*-Glycopeptides Using Multilayered Mesoporous Covalent Organic Polymers (Centrifuge-Based)

**Figure 3:**
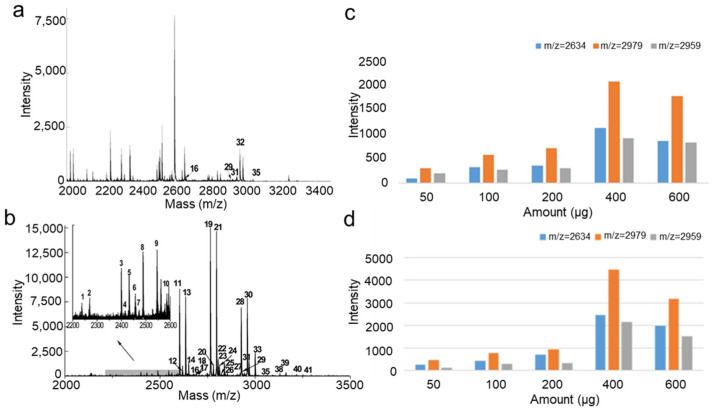
MALDI-TOF spectrum of IgG tryptic digest before enrichment (**a**), and after enrichment with p-TpBDH (**b**). Comparison of signal intensities of three glycopeptides enriched from 2 µg of tryptic IgG digest (**c**) after enrichment with p-TpBDH, (**d**) and after enrichment with p-TpBDH-OH.

Protein *N*-glycosylation is one of the most common and important PTMs, playing a critical role in regulating various physiological processes. Abnormal *N*-glycosylation is closely associated with a wide range of diseases. Therefore, developing highly selective and sensitive glycopeptide enrichment strategies is of great significance for biomarker discovery and disease diagnosis. Based on our previous study [32], we preliminarily verified the enrichment capability of the material for *N*-glycopeptides (Figure 3, Table S1). Without enrichment, due to severe signal suppression by a large number of non-glycopeptides in the tryptic digest, only five glycopeptides were detected with relatively weak signal intensities (Figure 3a). Following enrichment using a layered imine-based covalent organic polymer with mesopores (p-TpBDH) and its hydrophilic group-rich derivative (p-TpBDH-OH), a total of 36 and 40 glycopeptides were identified, respectively. The application of these materials significantly enhanced the detection of glycopeptides with higher signal-to-noise ratios, as shown in Figure 3b and Figure S1. These findings collectively highlight the excellent selectivity and enrichment performance of the synthesized materials for *N*-glycopeptides.

To experimentally validate the trend predicted by our theoretical model, varying amounts of enrichment material were applied to extract glycopeptides from 2 μg of IgG tryptic digest (1 mg/mL). Three glycopeptides with *m*/*z* values of 2634, 2797, and 2959 were selected as marker ions for evaluation. As shown in Figure 3c,d, the signal intensities of these glycopeptides increased progressively with the amount of material used and reached a maximum at 0.4 mg. Notably, the glycopeptide signal intensities obtained using p-TpBDH-OH were significantly higher than those obtained with p-TpBDH, indicating its superior performance in glycopeptide enrichment. These findings suggest that p-TpBDH-OH holds considerable promise for applications in glycoproteomics. Importantly, the enrichment efficiency curves for both materials followed a consistent trend with our theoretical prediction—showing an initially increasing and then decreasing pattern—further confirming the validity of the model.

**Figure 4:**
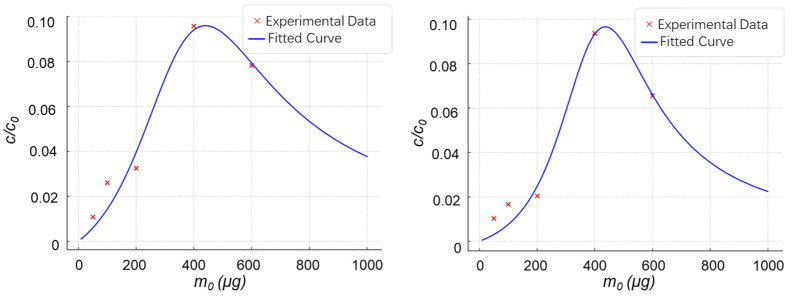
Fitted enrichment curves of p-TpBDH (**left**) and p-TpBDH-OH (**right**) materials for glycopeptides extracted from 2 µg of tryptic IgG digest.

Prior to establishing a formal theoretical framework, we empirically assumed that the optimal amount of enrichment material in a 200 μL solution system was approximately 400 μg. Accordingly, this material dosage was adopted in the subsequent experiments of that study. However, when experimental data were fitted into our theoretical model, the resulting relationship curves (Figure 4) provided refined insights. For the enrichment of *N*-glycopeptide with *m*/*z* 2797 using p-TpBDH, the fitted curve parameters were determined as *D* = 0.00514, *E* = 999.15, and *F* = −3.4912. From these, the calculated optimal material dosage corresponding to the peak of the curve was 440.72 μg (Figure 4, left). Similarly, for p-TpBDH-OH, the fitted curve parameters were *D* = 0.0108, *E* = 2054.62, and *F* = −8.3760, yielding an optimal enrichment dosage of 436.63 μg (Figure 4, right). These results not only validate our theoretical model but also demonstrate its utility in optimizing enrichment conditions with greater precision.

These findings provide initial validation that our theoretical framework can be effectively integrated with experimental observations for the enrichment of glycosylated peptides. The consistency between the model and empirical data highlights the model’s predictive capability and its utility in guiding the optimization of material dosage. This integration facilitates the rational design of enrichment protocols, ultimately enhancing the efficiency and specificity of glycopeptide enrichment.

#### 2.2.2. Functionalized Magnetic Covalent Organic Framework Materials for the Selective Enrichment of Phosphopeptides (Magnetic-Based)

To further substantiate the generalizability of our theoretical model beyond centrifugation-based systems and glycosylation-specific enrichment, we applied it to an independent enrichment platform, which we previously developed [33]. The selective enrichment of phosphorylated peptides using functionalized magnetic materials. This investigation aimed to assess whether the predicted enrichment trends hold true across different classes of PTMs, thereby demonstrating the broader applicability and robustness of the model.

**Figure 5:**
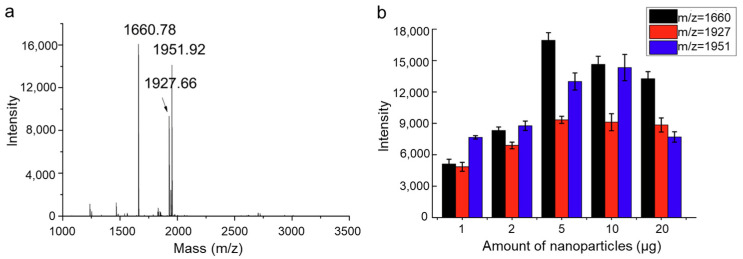
(**a**) Enrichment of α-Casein Tryptic Digests by Fe_3_O_4_@SiO_2_@TpPa-Ti^4+^ Magnetic Nanoparticles. (**b**) Detection of the Enrichment Performance for Three Characteristic Phosphopeptides from 1 µg of α-Casein Tryptic Digest.

To systematically evaluate the performance of Fe_3_O_4_@SiO_2_@TpPa-Ti^4+^ composite materials in phosphopeptide enrichment, tryptic digests of the standard phosphoprotein α-casein were employed as model samples. Three phosphopeptides with *m*/*z* values of 1660.8, 1927.7, and 1951.9 were selected as marker ions due to their strong signal intensities in mass spectrometric analysis (Figure 5a), indicating higher enrichment efficiency and, thus, providing a clearer representation of the material’s enrichment capability. Each material dosage was tested in triplicate biological replicates to confirm the reproducibility of the enrichment process. Notably, the differing *m*/*z* values correspond to distinct peptide sequences; therefore, monitoring the signal variations in these markers enables further investigation into the selectivity and adsorption behavior of Fe_3_O_4_@SiO_2_@TpPa-Ti^4+^ nanocomposites toward various phosphorylated peptides.

This study not only facilitates the optimization of material dosage but also provides valuable experimental insight for subsequent phosphoproteomic analyses. Based on the results, it can be concluded that the experiments exhibited excellent reproducibility. The results revealed that when the material dosage was increased to 5 µg, two of the phosphopeptides reached maximum enrichment efficiency, while the third peptide required a dosage of 10 µg to achieve optimal recovery (Figure 5b). This observation indicates that the enrichment capacity and adsorption efficiency of Fe_3_O_4_@SiO_2_@TpPa-Ti^4+^ nanoparticles vary with peptide sequence. These differences are likely governed by factors such as peptide structure, phosphorylation site location, and specific interactions between the material surface and target molecules.

**Figure 6:**
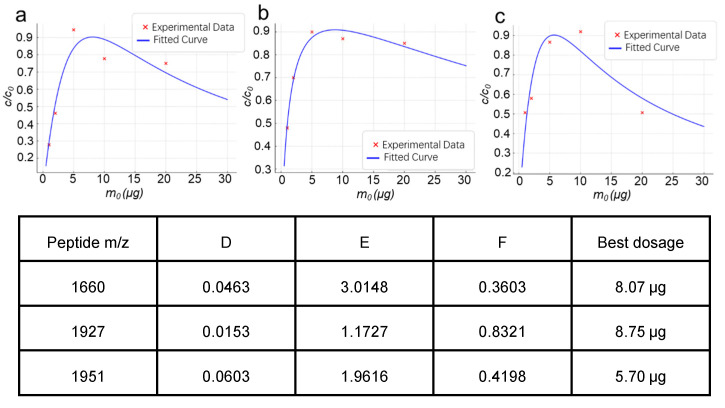
Fitted Enrichment Curves of Fe_3_O_4_@SiO_2_@TpPa-Ti^4+^ for Phosphopeptides with *m*/*z* 1660.8 (**a**), 1927.7 (**b**), and 1951.9 (**c**), Along with Corresponding Parameters and Optimal Material Dosages.

Similarly, by fitting the experimental data to the established model, we obtained enrichment curves for Fe_3_O_4_@SiO_2_@TpPa-Ti^4+^ magnetic nanoparticles targeting different phosphopeptides (Figure 6). From these fitted curves, the optimal material dosage corresponding to the maximum enrichment efficiency was calculated for each phosphopeptide. These results further support the applicability of our theoretical model in predicting optimal enrichment conditions and highlight the necessity of tailoring material dosages for specific phosphopeptide targets to achieve maximal recovery.

These experimental results clearly demonstrate that the enrichment efficiency of different phosphopeptides is significantly influenced by the amount of material used, and that each specific phosphopeptide exhibits a distinct optimal enrichment point. At this optimal point, the adsorption capacity of the enrichment material and the binding affinity for the phosphopeptide reach a dynamic equilibrium, resulting in the highest recovery rate and optimal signal detection performance.

## 3. Discussion

We began by constructing a physical model to describe the enrichment process of free beads in suspension systems, deriving a theoretical relationship between material dosage and target analyte concentration through thermodynamic analysis. By integrating this theoretical framework with experimental data, we systematically explored the application and optimization of enrichment materials for target molecule capture. This work highlighted the critical need for effective sample pretreatment of complex biological matrices prior to mass spectrometry analysis.

To evaluate the model’s applicability, enrichment performance was experimentally validated under both centrifugation-based and magnetic separation systems. These experiments focused on the selective enrichment of glycosylated and phosphorylated peptides, respectively. The thermodynamic and kinetic modeling revealed a non-linear relationship between the amount of enrichment material and the recovery of target analytes. The close agreement between theoretical predictions and empirical results further confirmed the robustness and accuracy of the model.

In the *N*-glycopeptide enrichment study, multilayered mesoporous covalent organic polymers (p-TpBDH and p-TpBDH-OH) were used to selectively enrich glycopeptides. Based on experimental feedback, the optimal material dosage was determined. The optimized conditions significantly improved enrichment efficiency by increasing glycopeptide recovery, reducing nonspecific adsorption, and enhancing signal-to-noise ratios. These findings provide preliminary validation of the theoretical model’s predictive power and practical utility.

In the phosphopeptide enrichment study, we synthesized a functionalized magnetic covalent organic framework, Fe_3_O_4_@SiO_2_@TpPa-Ti^4+^, and applied it to the enrichment of phosphopeptides derived from α-casein. The results revealed that enrichment efficiency was highly dependent on material dosage, and that each phosphopeptide exhibited a distinct optimal dosage point. At this point, the interaction between the phosphopeptide and the material surface reaches a dynamic equilibrium, yielding maximal recovery and detection sensitivity. Insufficient material leads to insufficient binding sites, limiting enrichment efficiency and causing loss of target phosphopeptides. Conversely, increasing the material dosage initially enhances peptide capture and signal strength. However, exceeding the optimal dosage does not further improve outcomes and may even compromise enrichment performance. Excess material can introduce nonspecific binding, reduce selectivity, saturate binding sites, and hinder the release of certain peptides. It may also increase background noise and co-enrichment of contaminants, thereby degrading mass spectrometric accuracy. Therefore, precise optimization of material dosage is crucial, not only to maximize recovery and sensitivity but also to reduce waste and ensure reproducibility. Additionally, the structural, charge-related, and conformational diversity among phosphopeptides leads to distinct interaction behaviors with enrichment materials. These differences influence the optimal material dosage for each peptide species, necessitating tailored optimization strategies. A systematic understanding of the relationship between material dosage and enrichment efficiency provides a solid experimental foundation for proteomic research. It also offers critical technical support for future quantitative phosphoproteomics and biomarker discovery, enabling more accurate and efficient analysis of phosphorylation events across diverse biological samples.

The results of this study demonstrate that the relationship between enrichment material dosage and enrichment efficiency is not linear; rather, there exists an optimal dosage. Deviations above or below this optimal point can negatively impact both enrichment efficiency and detection sensitivity. Therefore, careful optimization of material usage is essential to maximize the recovery of target molecules, enhance mass spectrometric signal intensity, reduce nonspecific adsorption, and improve data stability and reproducibility.

This work provides both theoretical insight and experimental evidence to support enrichment optimization strategies in the fields of proteomics and PTMs analysis. It establishes a solid foundation for the development of more efficient and precise bioanalytical mass spectrometry methods in future research.

## 4. Materials and Methods

### 4.1. Materials

Chemicals, unless otherwise specified, were from Sigma-Aldrich (St. Louis, MO, USA) or Thermo Fisher Scientific (Pittsburg, PA, USA). PNGase F was purchased from New England Biolabs (Ipswich, MA, USA). Acetonitrile, trifluoroacetic acid (TFA), and ammonium hydroxide (NH_3_·H_2_O) were of chromatographic grade and obtained from Aladdin (Shanghai, China).

### 4.2. Synthesis of Multilayered p-TpBDH and p-TpBDH-OH Materials

The synthesis of p-TpBDH was carried out in a 10 mL reaction system. Specifically, 0.45 mmol of benzene-1,2,4,5-tetracarboxylic dianhydride (BDH) and 0.3 mmol of 1,3,5-triformylphloroglucinol (Tp) were dissolved in a mixture containing 0.5 mL of 6 mol/L glacial acetic acid, 1.5 mL of dioxane, and 3 mL of *N*,*N*-dimethylacetamide (DMAc). The resulting suspension was ultrasonicated for 10 min to achieve a homogeneous dispersion, and then heated at 120 °C for 3 days. After the reaction, a light red insoluble powder was obtained and washed by ultrasonication with DMF to yield the layered p-TpBDH polymer. Subsequently, 1 mmol of p-TpBDH was reacted with 4 mmol of sodium borohydride (NaBH_4_) in 10 mL of ethanol at 0 °C for 12 h. The final product was a dark red p-TpBDH-OH powder.

### 4.3. Synthesis of Magnetic Fe_3_O_4_@SiO_2_@TpPa-Ti^4+^ Nanoparticles

In brief, magnetic Fe_3_O_4_ nanoparticles were synthesized via a solvothermal method, while Fe_3_O_4_@SiO_2_ nanoparticles were prepared using the Stöber sol-gel method according to a reported procedure [37]. The surface of the Fe_3_O_4_@SiO_2_ nanoparticles was subsequently functionalized with amino groups to obtain Fe_3_O_4_@SiO_2_-NH_2_. Next, Fe_3_O_4_@SiO_2_-NH_2_ was dispersed in a mixed solution of dioxane and 1,3,5-triformylphloroglucinol (Tp), and heated at 120 °C for 1 h to facilitate a condensation reaction between amine and aldehyde groups, yielding Fe_3_O_4_@SiO_2_-Tp nanoparticles. Under vacuum and in the presence of glacial acetic acid as a catalyst, a further condensation reaction between Tp and Pa-NO_2_ monomers was carried out to form a TpPa-NO_2_ covalent organic framework (COF) layer on the nanoparticle surface, resulting in Fe_3_O_4_@SiO_2_@TpPa-NO_2_ nanoparticles. The nitro groups (-NO_2_) on TpPa-NO_2_ were then reduced to amino groups (-NH_2_) using sodium borohydride, producing Fe_3_O_4_@SiO_2_@TpPa-NH_2_. During this step, imine (C=N) linkages were also reduced to secondary amines (C-NH), thereby enhancing the material’s structural stability. To introduce phosphate functionalities for metal ion coordination, Fe_3_O_4_@SiO_2_@TpPa-NH_2_ was phosphorylated by dispersing it in an acetonitrile solution containing phosphoryl chloride and 2,4,6-trimethylpyridine, yielding Fe_3_O_4_@SiO_2_@TpPa-PO_4_^3−^ nanoparticles. Finally, through the chelation of phosphate groups with titanium ions, the material was further functionalized by treating it with a Ti(SO_4_)_2_ aqueous solution to obtain Fe_3_O_4_@SiO_2_@TpPa-Ti^4+^ nanoparticles.

### 4.4. Enzymatic Digestion of Standard Proteins

A total of 1 mg of IgG or α-Casein was dissolved in 100 mL of 50 mmol/L ammonium bicarbonate solution containing 8 mol/L urea to achieve full denaturation. Then, 0.15 mg of dithiothreitol (DTT) was added, and the mixture was incubated at 60 °C for 40 min to reduce disulfide bonds between cysteine residues. Subsequently, 0.72 mg of iodoacetamide (IAA) was added to neutralize the excess DTT, and the solution was incubated in the dark for 30 min to alkylate the cysteine residues. Finally, trypsin was added at a mass ratio of 1:25 (enzyme/protein, *w*/*w*), and the mixture was incubated at 37 °C for 17 h to complete digestion.

### 4.5. Enrichment of N-Glycopeptides

The IgG digest was first diluted to the desired concentration using a loading buffer. A defined amount of p-TpBDH-OH material was then added to 200 µL of the diluted sample and incubated at room temperature for 30 min to allow sufficient adsorption of glycopeptides onto the material surface. After incubation, the supernatant was removed by centrifugation. The p-TpBDH-OH material was subsequently washed three times with loading buffer to eliminate nonspecifically bound peptides, thereby ensuring optimal enrichment selectivity. Finally, glycopeptides were eluted from the nanomaterial using 200 µL of elution buffer (5% trifluoroacetic acid in 30% acetonitrile/water). The eluate was dried under vacuum and reconstituted in 20 µL of the same elution buffer. The resulting solution was ready for MALDI-TOF-MS (matrix-assisted laser desorption/ionization time-of-flight mass spectrometry) analysis.

### 4.6. Enrichment of Phosphopeptides

To enrich phosphopeptides from the tryptic digest of the standard phosphoprotein α-casein, 10 µg of Fe_3_O_4_@SiO_2_@TpPa-Ti^4+^ magnetic nanoparticles was added to 200 µL of loading buffer (50% acetonitrile, 1% trifluoroacetic acid) containing 15 pmol of α-casein digest. The mixture was incubated at room temperature for 30 min to allow selective binding of phosphopeptides. After incubation, the nanoparticles were collected using a magnet and washed three times with 200 µL of washing buffer (50% acetonitrile, 1% TFA) to remove nonspecifically adsorbed peptides. Finally, phosphopeptides were eluted by incubating the material with 10 µL of 10 wt% ammonium hydroxide for 10 min. The eluate was collected and subjected to MALDI-TOF-MS analysis.

### 4.7. MALDI-TOF/TOF Mass Spectrometry Analysis

All MALDI-TOF MS analyses were performed using a 5800 MALDI-TOF/TOF mass spectrometer (AB Sciex, USA) equipped with a Nd:YAG laser (wavelength: 355 nm). During the experiment, 0.5 µL of the eluate was spotted onto the MALDI target plate and air-dried at room temperature. Subsequently, 0.5 µL of matrix solution (20 mg/mL 2,5-dihydroxybenzoic acid, DHB, in ACN/H_2_O/H_3_PO_4_ = 70:29:1, *v*/*v*) was added and dried again before analysis. The instrument was operated with a laser pulse frequency of 200 Hz and an acceleration voltage of 20 kV.

## 5. Conclusions

In this study, we developed a thermodynamically grounded physical model to describe the relationship between enrichment material dosage and target analyte in suspension enrichment systems. By integrating theoretical derivations with experimental validation, we established a quantitative framework for optimizing enrichment conditions in both centrifugation-based and magnetic-based workflows. Our model revealed a non-linear relationship between material dosage and enrichment efficiency, characterized by a defined optimal dosage that maximizes recovery and signal intensity while minimizing nonspecific adsorption and analytical interference.

Our main purpose was to calculate the appropriate dose to enrich PTM peptides using a thermodynamics-based model, and this was demonstrated by applying it to two types of PTM samples. We experimentally validated this model in two representative PTMs systems: the selective enrichment of *N*-glycopeptides using multilayered mesoporous covalent organic polymers (p-TpBDH and p-TpBDH-OH), and the enrichment of phosphopeptides using a functionalized magnetic covalent organic framework (Fe_3_O_4_@SiO_2_@TpPa-Ti^4+^). In both systems, the predicted enrichment trends were in excellent agreement with experimental observations, demonstrating the model’s predictive accuracy and broad applicability.

Our findings underscore the critical importance of rational dosage optimization in improving the specificity, sensitivity, and overall performance of enrichment-based proteomic workflows. This work provides a robust theoretical and experimental foundation for enhancing enrichment strategies in PTMs analysis and supports the development of more sensitive, precise, and efficient mass spectrometry-based proteomics platforms.

## Data Availability

The original contributions presented in this study are included in the article. Further inquiries can be directed to the corresponding authors.

## Supplementary Materials

The following supporting information can be downloaded at: https://www.mdpi.com/article/10.3390/molecules30153245/s1, Figure S1: MALDI-TOF Mass Spectrum of IgG (1.67 × 10 ^−7^ mol/L, 200 µL) Tryptic Digest After Enrichment with p-TpBDH-OH. Table S1. Detailed characterization of glycopeptides enriched from IgG tryptic digest using p-TpBDH and p-TpBDH-OH materials.
