# Supplementary material for: Theoretical Investigation of the Material Usage During On-Bead Enrichment of Post-Translationally Modified Peptides in Suspension Systems
Source: Molecules. 2025 Aug 2;30(15):3245. doi: 10.3390/molecules30153245 (PMC12348116; doi:10.3390/molecules30153245)
Supplement: Supplementary file 1 [file molecules-30-03245-s001.zip › molecules-3753574-supplementary.pdf]

Theoretical Investigation of the Material Usage During On-Bead Enrichment of Post-Translationally Modified Peptides in Suspension Systems

Kai Liu 1, Yuanyu Huang 1, Thomas Huang 2, Pengyuan Yang 1,3, Jilie Kong 1, Huali Shen 3,* and Quanqing Zhang 1,2,*

1 Department of Chemistry, Fudan University, Shanghai 200433, China; 20110220123@fudan.edu.cn (K.L.); 18110220040@fudan.edu.cn (Y.H.); pyyang@fudan.edu.cn (P.Y.); jlkong@fudan.edu.cn (J.K.)

2 Institute for Integrative Genome Biology, Proteomics Core, University of California Riverside, Riverside, CA 92521, USA; thomas.huang001@email.ucr.edu

3 Institutes of Biomedical Sciences of Shanghai Medical School, Fudan University, Shanghai 200032, China

* Correspondence: shenhuali@fudan.edu.cn (H.S.); quanqinz@ucr.edu (Q.Z.)

Table of contents

Figure S1. MALDI-TOF Mass Spectrum of IgG (1.67 × 10^{-7} mol/L, 200 μL) Tryptic Digest After Enrichment with p-TpBDH-OH

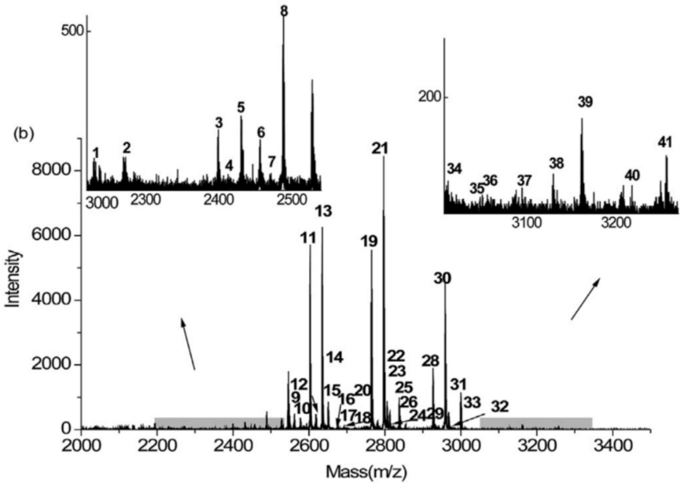

Table S1. Detailed characterization of glycopeptides enriched from IgG tryptic digest using p-TpBDH and p-TpBDH-OH materials.

N# denotes the identided N-linked glycosylation site. The mass tolerance were set as 20 ppm.

No.	m/z	Glycan composition	Glycopeptide sequence	Enriched by TpBDH	Enriched by TpBDH-OH
1	2237.90	[Hex]3[HexNAc]2[Fuc]1	EEQFN#STFR	√	√
2	2268.80	[Hex]3[HexNAc]2[Fuc]1	EEQYN#STYR	√	√
3	2399.90	[Hex]3[HexNAc]3[Fuc]1	EEQFN#STFR	√	√
4	2413.90	[Hex]3[HexNAc]3[Fuc]1	EEQFN#STYR	√	√
5	2430.86	[Hex]3[HexNAc]3[Fuc]1	EEQYN#STYR	√	√
6	2456.87	[Hex]3[HexNAc]4	EEQFN#STFR	√	√
7	2470.97	[Hex]3[HexNAc]4	EEQFN#STYR	√	√
8	2487.89	[Hex]3[HexNAc]4	EEQYN#STYR	√	√
9	2561.00	[Hex]4[HexNAc]3[Fuc]1	EEQFN#STFR	√	√
10	2593.93	[Hex]4[HexNAc]3[Fuc]1	EEQYN#STYR	√	√
11	2601.98	[Hex]3[HexNAc]4[Fuc]1	EEQFN#STFR	√	√
12	2617.97	[Hex]4[HexNAc]4	EEQFN#STFR	√	√
13	2633.96	[Hex]3[HexNAc]4[Fuc]1 or [Hex]4[HexNAc]4	EEQYN#STYR or EEQFN#STYR	√	√
14	2649.96	[Hex]3[HexNAc]4[Fuc]1	EEQYN#STYR	√	√
15	2661.08	[Hex]3[HexNAc]5	EEQFN#STFR	×	√
16	2672.97	[Hex]3[HexNAc]5	EEQFN#STYR	√	√
17	2676.96	[Hex]3[HexNAc]5	EEQYN#STYR	√	√
18	2690.98	[Hex]3[HexNAc]5	EEQYN#STYR	√	√
19	2764.03	[Hex]4[HexNAc]4[Fuc]1	EEQFN#STFR	√	√
20	2781.01	[Hex]5[HexNAc]4 or [Hex]4[HexNAc]4[Fuc]1	EEQFN#STFR or EEQFN#STYR	√	√
21	2796.99	[Hex]4[HexNAc]4[Fuc]1 or [Hex]5[HexNAc]4	EEQYN#STYR or EEQFN#STYR	√	√
22	2805.05	[Hex]3[HexNAc]5[Fuc]1	EEQFN#STFR	√	√
23	2811.99	[Hex]5[HexNAc]4	EEQYN#STYR	√	√
24	2822.03	[Hex]4[HexNAc]5	EEQFN#STFR	√	√
25	2837.01	[Hex]3[HexNAc]5[Fuc]1 or [Hex]4[HexNAc]5	EEQYN#STYR or EEQFN#STYR	√	√
26	2853.10	[Hex]4[HexNAc]5	EEQYN#STYR	√	√
27	2909.03	[Hex]4[HexNAc]4 [NeuAc]1	EEQFN#STFR	√	×
28	2926.08	[Hex]5[HexNAc]4[Fuc]1	EEQFN#STFR	√	√

29	2943.03	[Hex]5[HexNAc]4[Fuc]1	EEQFN#STYR	√	√
30	2959.10	[Hex]5[HexNAc]4[Fuc]1	EEQYN#STYR	√	√
31	2967.10	[Hex]4[HexNAc]5[Fuc]1	EEQFN#STFR	√	√
32	2983.06	[Hex]5[HexNAc]5 or [Hex]4[HexNAc]5[Fuc]1	EEQFN#STFR	×	√
33	3000.07	[Hex]4[HexNAc]5[Fuc]1 or [Hex]5[HexNAc]5	EEQYN#STYR or EEQFN#STYR	√	√
34	3015.09	[Hex]5[HexNAc]5	EEQYN#STYR	×	√
35	3057.09	[Hex]4[HexNAc]4[Fuc]1[NeuAc]1	EEQFN#STFR	√	√
36	3088.06	[Hex]4[HexNAc]4[Fuc]1[NeuAc]1	EEQYN#STFR	×	√
37	3104.10	[Hex]5[HexNAc]4 [NeuAc]1	EEQYN#STYR	×	√
38	3129.13	[Hex]5[HexNAc]5[Fuc]1	EEQFN#STFR	√	√
39	3161.10	[Hex]5[HexNAc]5[Fuc]1	EEQYN#STYR	√	√
40	3217.15	[Hex]5[HexNAc]4[Fuc]1[NeuAc]1	EEQFN#STFR	√	√
41	3249.14	[Hex]5[HexNAc]4[Fuc]1[NeuAc]1	EEQFN#STFR	√	√

√ : Identified glycopeptides; ×: Non-identified glycopeptides.
